# Periodontal and dental effects of surgically assisted rapid maxillary expansion, assessed by using digital study models 

**DOI:** 10.1590/2176-9451.20.3.058-063.oar

**Published:** 2015

**Authors:** Danilo Furquim Siqueira, Mauricio de Almeida Cardoso, Leopoldino Capelozza, Dov Charles Goldenberg, Mariana dos Santos Fernandes

**Affiliations:** 1 Coordinator of the Postgraduate course in Orthodontics, Sociedade Paulista de Ortodontia, Botucatu, São Paulo, Brazil; 2 Professor of Orthodontics, Universidade Sagrado Coração (USC), Bauru, São Paulo, Brazil; 3 Full professor, Universidade de São Paulo (USP), School of Medicine, Department of Surgery, São Paulo, São Paulo, Brazil; 4 MSc in Orthodontics, Universidade Metodista de São Paulo (UMESP), São Bernardo do Campo, São Paulo, Brazil

**Keywords:** Orthodontics, Periodontics, Palatal expansion technique, Dental casts

## Abstract

**OBJECTIVE:**

The present study assessed the maxillary dental arch changes produced by surgically assisted rapid maxillary expansion (SARME).

**METHODS:**

Dental casts from 18 patients (mean age of 23.3 years) were obtained at treatment onset (T_1_), three months after SARME (T_2_) and 6 months after expansion (T_3_). The casts were scanned in a 3D scanner (D-250, 3Shape, Copenhagen, Denmark). Maxillary dental arch width, dental crown tipping and height were measured and assessed by ANOVA and Tukey's test.

**RESULTS:**

Increased transversal widths from T_1_ and T_2_ and the maintenance of these values from T_2_ and T_3_ were observed. Buccal teeth tipping also showed statistically significant differences, with an increase in all teeth from T_1_ to T_2 _and a decrease from T_2_ to T_3_. No statistically significant difference was found for dental crown height, except for left first and second molars, although clinically irrelevant.

**CONCLUSION:**

SARME proved to be an effective and stable procedure, with minimum periodontal hazards.

## Abstract (pt)

**OBJETIVOS:**

o presente estudo teve o objetivo de avaliar as alterações dentárias e periodontais decorrentes da Expansão Rápida da Maxila Assistida Cirurgicamente (ERMAC).

**MÉTODOS:**

foram obtidos os modelos de gesso de 18 pacientes (média de idade de 23,3 anos), ao início (T_1_), 3 meses após a ERMAC (T_2_) e 6 meses após a expansão (T_3_). Os modelos foram digitalizados (Scanner 3D 3Shape D-250) e mensuraram-se as distâncias transversais, bem como a inclinação e a altura da coroa clínica dos dentes posteriores. Para análise dos resultados, aplicou-se a análise de Variância e o teste de Tukey.

**RESULTADOS:**

nas distâncias transversais, observou-se um aumento de T_1_ para T_2_ e uma manutenção de T_2_ para T_3_. As inclinações dentárias demonstraram diferenças estatisticamente significativas em alguns dentes; porém, numericamente tenderam a um aumento de T_1_ para T_2_ e a uma diminuição de T_2_ para T_3_. Não se observou diferença estatisticamente significativa na altura da coroa clínica, exceto nos primeiros e segundos molares do lado esquerdo, porém, clinicamente irrelevante.

**CONCLUSÕES:**

a ERMAC demonstrou ser um procedimento efetivo e estável, com mínima repercussão periodontal.

## INTRODUCTION

Proper maxillary transverse dimension is a key component of optimal, stable occlusion. Rapid maxillary expansion (RME) is a procedure commonly employed by orthodontists treating transverse issues.^1^^-^ 5 Despite being successful in children and adolescents, this procedure fails when performed in patients in the final growth phase and in adults.^1^^,^ 2 ^,^ 6 ^,^ 7 ^,^ 8

After growth ends, the amount of force required to split the midpalatal suture is relatively high due to increases both in the complexity of this suture and in the rigidity of adjacent facial structures. Thus, enlarging the maxillary complex by nonsurgical expansion in adults can cause side effects, such as higher relapse rates, tipping of support teeth, severe pain and gingival recession,^1^^,^ 2 ^,^ 6 ^,^ 9 since the forces delivered during expansion may produce buccal tipping of teeth, thereby generating areas of compression in the periodontal ligament of support teeth.^10^^,^ 11 In these cases, midpalatal suture splitting must be combined with a surgical procedure known as surgically assisted rapid maxillary expansion (SARME) which breaks down sutural resistance and enables maxillary expansion without the aforementioned side effects.^1^^,^ 3 ^,^ 4 ^,^ 6 ^,^ 9 ^,^ 12 ^,^ 13

The benefits of treating transverse maxillary deficiency include improvements in dental and skeletal stability, decreased need for extractions to perform alignment and leveling, increased teeth visibility at smiling, and, occasionally, improvements in nasal breathing.^5^^,^ 12 ^,^ 14 ^,^ 15

There are numerous ways to assess changes resulting from SARME, but in the last two decades, thanks to remarkable technological advances in Dentistry, cutting edge analysis tools have emerged. In Orthodontics, these advances have primarily occurred in diagnostic elements, such as the use of photography and digital radiography. The use of digital dental casts was introduced by the orthodontic industry as a component of the new, now fully digitized and highly accurate orthodontic records.^7^^,^ 16 ^-^ 23 Thus, this study aims at analyzing, with the aid of digital models, the major changes produced in the transverse dimension and tipping of maxillary teeth, as well as the potential impact of this procedure on adult patients undergoing SARME. 

## MATERIAL AND METHODS

This project was submitted to Universidade Metodista de São Paulo Institutional Review Board, and approved under protocol number 142.170/07.

This is a retrospective study of which sample comprised 54 maxillary dental casts obtained from 18 adult patients with maxillary atresia, 6 men and 12 women, with a mean age of 23.3 years (minimum of 18 and maximum of 35 years old) from the Postgraduate Clinic of Universidade Metodista de São Paulo. All subjects underwent SARME.

To perform the expansion procedure, a 13-mm Hyrax expansion screw was used.^24^ Moreover, a conservative surgical technique consisting of LeFort I osteotomy was employed to approach the midpalatal suture without involving the pterygopalatine suture.^25^ All surgeries were conducted by the same surgeon.

The expansion screw was first activated on the third day after surgery, and patients were instructed to make two daily activations, one in the morning (1/4 turn) and one at night (1/4 turn), until the screw was fully opened, or until it reached the desired overcorrection (palatal cusp of the maxillary first molar edge-to-edge with the buccal cusp of the mandibular first molar).

The appliance (Hyrax) remained in the oral cavity for three months, functioning as a retainer. After this period, the expander was removed and an acrylic plate (with retention clips between premolars) was inserted and remained in place for three months until a fixed orthodontic appliance was placed.

For variables assessment, dental casts were scanned with a 3D scanner (D-250, 3Shape, Copenhagen, Denmark). Only the maxillary models during phases T_1_ (initial), T_2_ (three months post-expansion) and T_3_ (six months post-expansion) were used.

**Figure 1.**
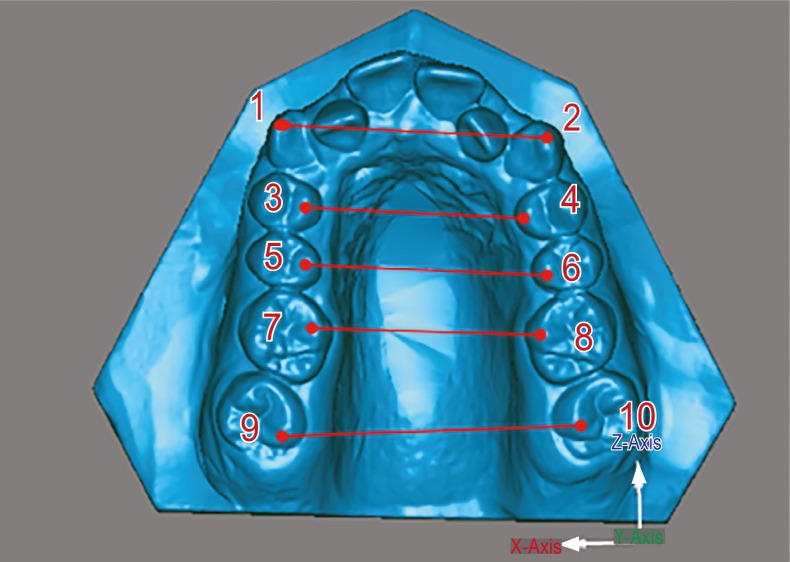
Points and transverse widths in the digital models: 1) Cusp tip of right canine; 2) Cusp tip of left canine; 3) Palatal cusp tip of right maxillary first premolar; 4) Palatal cusp tip of left maxillary first premolar; 5) Palatal cusp tip of right maxillary second premolar; 6) Palatal cusp tip of left maxillary second premolar; 7) Mesio-palatal cusp tip of right maxillary first molar; 8) Mesio-palatal cusp tip of left maxillary first molar; 9) Mesio-palatal cusp tip of right maxillary second molar; 10) Mesio-palatal cusp tip of left maxillary second molar.

Linear measurements were taken by means of Geomagic Studio 5^TM^ (Research Triangle Park, USA), a software that allows viewing and manipulating digital representations on a computer screen. Transverse changes resulting from SARME were assessed by means of intercanine, interpremolar and intermolar widths (Fig 1), using the points described by Currier^26^ and Berger et al^27^ as reference.

**Figure 2.**
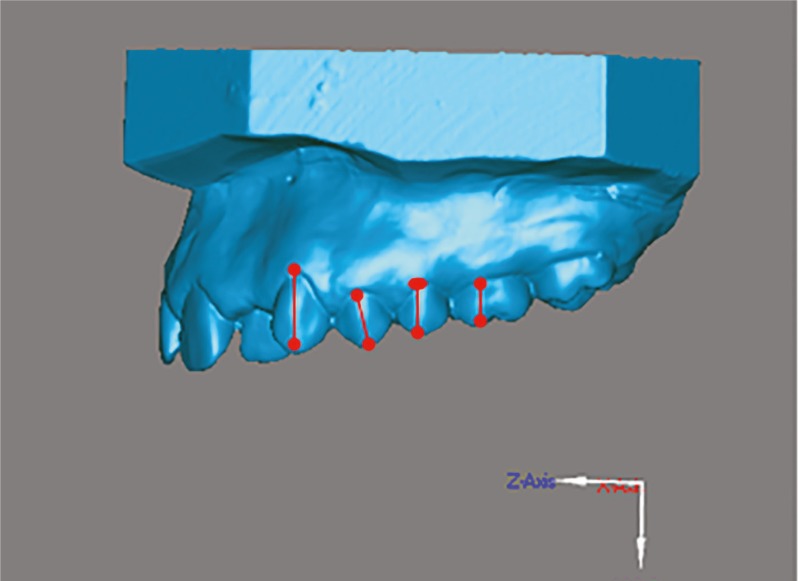
Height of clinical crowns.

The height of the clinical crown of canines, premolars and molars was measured based on the distance between the buccal cusp and the most apical point of the gingival margin,^5^^,^ 9 as shown in Figure 2.

**Figure 3.**
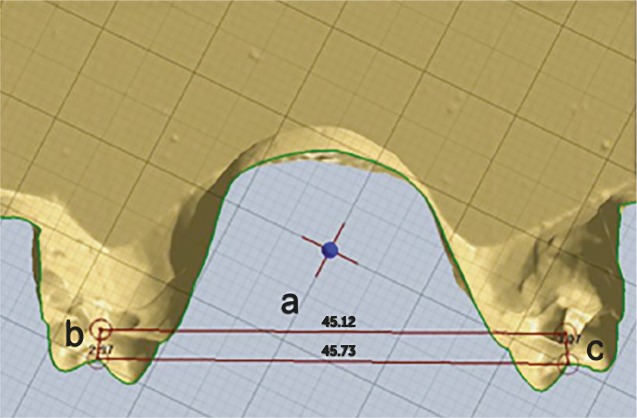
Defining lines a, b and c

Angular measurements were taken with the aid of OrthoDesigner^TM^ software (3Shape, Copenhagen, Denmark) which also features tools to assist in obtaining angular measurements and slicing dental casts. Intercanine, interpremolar and intermolar tipping was calculated using the following references^5^: Line a= distance between the left and right midpoints of the deepest region of buccal and palatal surfaces in the gingival margin; Line b= distance between the geometric midpoint on the right side of the center of buccal and palatal cusps, and the midpoint of the deepest region in the gingival margin; Line c= distance from the left side of the geometric midpoint at the center of buccal and palatal cusps, and the midpoints of the deepest buccal and palatal portions of the gingival margin. With these reference lines, the internal angles formed by lines a-b and a-c were calculated with the aid of the software. After this definition, the bilateral angulation of posterior teeth was calculated (Fig 3).

**Figure 4.**
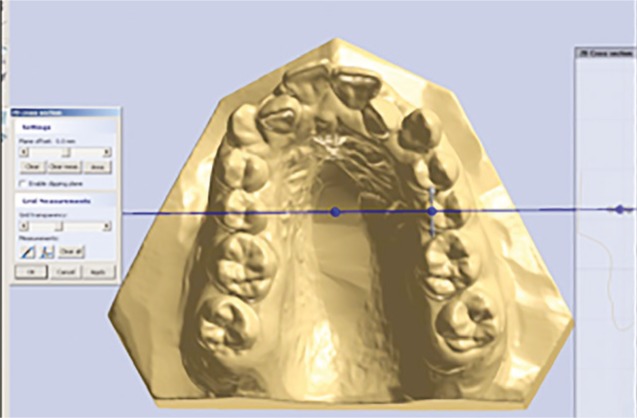
Defining clipping plane.

To this end, it was necessary to create a clipping plane in the models (Fig 4) to allow teeth to be viewed mesially. The reference plane met the aforementioned criteria.

**Figure 5.**
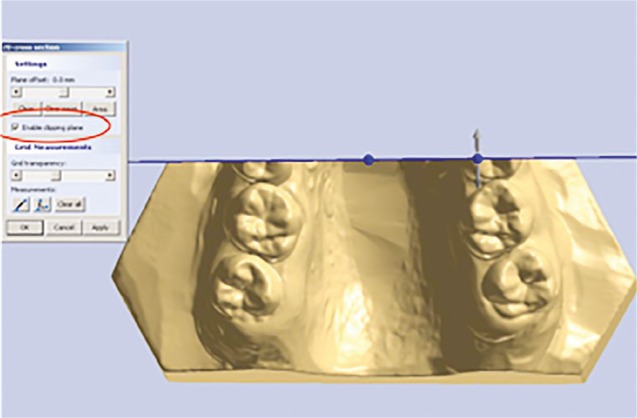
Enabling clipping plane tool, shown in red.

In selecting the clipping plane, the tool "enable clipping plane" was used. This allowed the mesial viewing of the models, as it excluded their anterior portion (Fig 5). The changes in each parameter occurring during treatment were calculated in the models at the times described before.

## Statistical analysis

To determine the error of the method, 30% of the sample was randomly selected and measured after at least one week, using the same material and applying the same aforementioned criteria. Paired t-test was used to determine intraexaminer systematic error. Random error was calculated by Dahlberg's formula.^28^

In order to compare the three assessment periods, analysis of variance (ANOVA) was used with a criterion for repeated measurements. When ANOVA revealed statistically significant difference, Tukey's test for multiple comparisons was applied. A level of significance of 5% (p < 0.05) was adopted for all tests.

## RESULTS

From the foregoing, one can argue that the results found in this study are reliable, since, after further measurements were carried out in the dental casts of five randomly selected patients, no intraexaminer errors that might compromise this research were identified. Measurements of tooth tipping are more error-prone due to inconsistencies in (a) the location of points, (b) trimming of casts, and (c) construction of lines.

Table 1 depicts means and standard deviation values of transverse widths in the upper dental arch, expressed in millimeters, at the three evaluation periods, and results from ANOVA and Tukey's test. It shows an increase in transverse width with means of 9.26 mm for first molars, 5.4 mm for second molars, 9.8 mm for first premolars, 9.49 mm for second premolars, and 5.87 mm for canines from T_1_ to T_2_. These values remained unchanged from T_2_ to T_3_.

**Table 1.** Means and standard deviation values of transverse widths in the upper dental arch, in mm, at the three assessment periods, and results of ANOVA and Tukey's test.

Tooth	T_1_	T_2_	T_3_	p
1^st^M	35.94 ± 4.43^a^	45.20 ± 3.96^b^	45.26 ± 4.41^b^	< 0.001*
2^nd^M	43.45 ± 4.58^a^	48.85 ± 4.53^b^	48.82 ± 4.72^b^	< 0.001*
1^st^PM	26.02 ± 2.56^a^	35.82 ± 2.97^b^	35.47 ± 2.69^b^	< 0.001*
2^nd^PM	31.28 ± 3.24^a^	40.77 ± 3.05^b^	40.73 ± 3.20^b^	< 0.001*
C	30.56 ± 2.39^a^	36.43 ± 2.41^b^	36.14 ± 2.57^b^	< 0.001*

* Statistically significant difference found by ANOVA (p < 0.05). Periods of time with the same letter are not statistically different (Tukey's test).

Table 2 presents the mean size of crowns in the maxillary arch, expressed in millimeters, at T_1_, T_2_ and T_3_, and the results of ANOVA and Tukey's test showing differences only in left first and second molars.

**Table 2.** Means and standard deviation values of crown heights in the upper dental arch, in mm, at the three assessment periods, and results of ANOVA and Tukey's test.

Tooth	T_1_	T_2_	T_3_	p
1^st^M right	7.25 ± 1.08	7.25 ± 0.84	7.23 ± 0.81	0.996 ns
2^nd^M right	6.88 ± 1.00	7.19 ± 0.78	7.05 ± 0.79	0.182 ns
1^st^PM right	7.66 ± 0.89	7.92 ± 0.94	8.04 ± 0.88	0.062 ns
2^nd^PM right	6.88 ± 1.11	6.88 ± 1.15	6.98 ± 0.94	0.754 ns
C right	9.29 ± 1.15	9.47 ± 1.14	9.40 ± 0.98	0.492 ns
1^st^M left	6.96 ± 0.74^a^	7.17 ± 1.03^ab^	7.39 ± 1.11^b^	0.035*
2^nd^M left	6.45 ± 0.78^a^	6.89 ± 0.89^b^	6.87 ± 0.91^b^	0.006*
1^st^PM left	7.92 ± 1.03	8.00 ± 0.79	8.03 ± 0.70	0.748 ns
2^nd^PM left	6.81 ± 0.99	6.84 ± 0.93	6.84 ± 0.97	0.930 ns
C left	9.21 ± 1.00	9.35 ± 1.15	9.38 ± 0.99	0.597 ns

* Statistically significant difference found by ANOVA (p < 0.05). Periods of time with the same letter are not statistically different (Tukey's test).

Table 3 shows means and standard deviation values of maxillary teeth tipping, expressed in degrees, at T_1_, T_2_and T_3_, and the results of ANOVA and Tukey's test. All values increased, thereby pointing to buccal tipping, although significant only in some teeth.

**Table 3.** Means and standard deviation values of crown heights in the upper dental arch, in mm, at the three assessment periods, and results of ANOVA and Tukey's test.

Tooth	T_1_	T_2_	T_3_	p
1^st^M right	98.96 ± 9.47	102.76 ± 5.63	102.61 ± 4.90	0.120 ns
2^nd^M right	106.98 ± 7.50^a^	110.82 ± 8.73^b^	109.41 ± 7.69^ab^	0.041*
1^st^PM right	88.18 ± 8.00^a^	96.37 ± 8.19^b^	95.48 ± 7.15^b^	< 0.001*
2^nd^PM right	92.20 ± 8.85^a^	101.54 ± 8.77^b^	100.34 ± 5.48^b^	< 0.001*
C right	100.17 ± 8.77	99.33 ± 8.11	98.76 ± 8.95	0.644 ns
1^st^M left	102.07 ± 7.10^a^	106.40 ± 7.46^b^	104.62 ± 5.72^ab^	0.019*
2^nd^M left	112.18 ± 6.28	113.70 ± 6.05	110.47 ± 6.34	0.154 ns
1^st^PM left	94.24 ± 6.53	96.17 ± 7.13	96.68 ± 6.30	0.312 ns
2PM left	93.33 ± 6.37^a^	103.84 ± 5.78^b^	101.45 ± 6.70 ^b^	< 0.001*
C left	103.29 ± 7.17	104.06 ± 7.84	100.17 ± 5.13	0.084 ns

ns = No statistically significant difference.

* Statistically significant difference found by ANOVA (p < 0.05). Periods of time with the same letter are not statistically different (Tukey's test).

## DISCUSSION

The literature presents different methods to assess changes induced by SARME in dental casts, namely: assessment with a bow compass,^27^digital calipter^4^ and laser-scanned models. Laser scanning is common in industrial engineering and medicine as a noninvasive alternative to generate 3D images. The measurement method using a 3D scanner has been studied and proved reliable and convenient.^7^^,^ 18 ^,^ 21 It has also been proven that analyses in digital models can be performed in both clinical practice and research, with extremely accurate outcomes.^16^^,^ 17 ^,^ 19 ^,^ 20

Digital models have the added advantage of allowing images to be sliced, providing superior viewing of points not visible in dental casts. Furthermore, they can be superimposed, which facilitates viewing of the mechanics used in a given treatment.^21^

The time spent while taking measurements in the digital models was relatively shorter, given the user-friendliness of the programs and the measuring resources available, which yield very accurate measurements.^23^

Treatment including SARME proved successful for adult patients requiring maxillary expansion, a finding reported by several authors.^2^^,^ 4 ^,^ 6 ^,^ 12 ^,^ 13 ^,^ 25

The present study disclosed an increase in transverse width in all teeth from T_1_ to T_2_, with measurements remaining unchanged from T_2_ to T_3_ (Table 1). Thus, it is reasonable to assert that SARME demonstrated effectiveness and stability during the assessment period (6 months).

The slight increase found in intercanine width can be attributed to the fact that patients with indication for SARME often have canines in infralabioversion. As anterior space is gained, these teeth tend to align, consequently taking on a more lingual position and not showing so much increase in width.^1^^,^ 4 ^,^ 13 ^,^ 27

In comparison to first molars, there was less increase in transverse width in second molars (5.4 mm and 9.4 mm, respectively). This difference can be probably linked to release of the pterygopalatine process due to the surgical technique employed, and also to the fact that this tooth was not included in the appliance.^25^

In adults, both surgical and nonsurgical procedures can correct maxillary transverse deficiency and achieve stability,^4^^,^ 5 ^,^ 8 ^,^ 9 but comparison showed greater transverse increase in surgical cases.

SARME did not interfere in gingival attachment at the three assessment periods, except for first and second molars on the left side. Bassareli, Dalstra and Melsen^5^ as well as Handelman et al^8^ reported that nonsurgical maxillary expansion is effective in adults. However, these studies demonstrated greater dentoalveolar compensation due to increased tipping. Furthermore, they found no connection between the development of gingival recession and the amount of transverse expansion in adults, since there was no change in clinical crown height. In comparing the two types of treatment, i.e., SARME *versus* nonsurgical expansion, Carmen et al^9^ found that these treatment modalities result in increased transverse dimension and show no statistically significant differences in the development of gingival recession. Nevertheless, SARME proved more effective and less harmful to the periodontium, thereby corroborating Northway and Meade,^4^ who argued that crown length displayed greater increase in nonsurgical patients.

The literature has shown that bone dehiscence can be produced in the alveolar bone when teeth are tipped bucally, but orthodontic movement would not necessarily be accompanied by loss of connective tissue.^10^^,^ 11

It has been acknowledged that teeth positioned or moved bucally, bone dehiscence and the presence of thin and brittle keratinized mucosa are the main predisposing factors of gingival recession.^15^^,^ 29 Gingival recession, however, is only triggered by mechanical trauma caused by brushing, or inflammation induced by the presence of plaque.^15^ Therefore, the quality of the keratinized mucosa and tooth brushing in particular should be closely monitored in patients undergoing SARME.

The surgical procedure resulted in dentoalveolar tipping, with statistical significance (Table 3), in the second molar, first and second premolars on the right side, and first molar and second premolars on the left side from T_1_ to T_2_. From T_2_ to T_3_, tipping remained unchanged. In this study, crown tipping was calculated by means of the angle formed by the long axis of the tooth with a line that connects the buccal and lingual surfaces of the gingival most points. Thus, calculating tipping was less dependent on crown morphology,^5^ since other methods are influenced by changes in cusp height.^1^^,^ 4

This difference in the amount of tipping may be related to the way in which expansive force is delivered. Second premolars experienced expansion forces through contact between the lingual connection wire and its homonymous surface. With simple force applied to the crown, away from the center of resistance, a moment of force was created in the buccal direction, ultimately yielding some tipping component. Furthermore, anchorage teeth received expansion forces by means of bands rigidly fixed to the appliance. As the screw was activated, the bands, which were wide in the cervico-occlusal direction, resisted the tendency to tip by moving the anchorage teeth predominantly through a bodily movement in buccal direction.^15^ This clearly shows that overcorrection was necessary due to relapse induced by the effects of tipping.^3^^,^ 4 ^,^ 8

## CONCLUSION

SARME proved to be an effective and stable procedure, with minimum periodontal hazards.
